# Complementary and alternative medicine modalities used to treat adverse effects of anti-cancer treatment among children and young adults: a systematic review and meta-analysis of randomized controlled trials

**DOI:** 10.1186/s12906-022-03537-w

**Published:** 2022-04-02

**Authors:** Dana C. Mora, Grete Overvåg, Miek C. Jong, Agnete E. Kristoffersen, Debbie C. Stavleu, Jianping Liu, Trine Stub

**Affiliations:** 1 Department of Community Medicine, Faculty of Health Science, National Research Center in Complementary and Alternative Medicine, NAFKAM, UiT The Arctic University of Norway, 9037 Tromsø, Norway; 2 Science and Health Library, UiT, The Arctic University of Norway, Hansine Hansens veg 19, 9019 Tromsø, Norway; 3 Princess Máxima Center for Pediatric Oncology, Utrecht, the Netherlands; 4 Centre for Evidence-Based Chinese Medicine, Beijing University of Chinese Medicine and Pharmacology, Beijing, China

**Keywords:** Complementary and alternative medicine (CAM), Pediatric oncology, Adverse effects, Chemotherapy-induced nausea and vomiting (CINV)

## Abstract

**Background:**

Dealing with the symptom burden of cancer diagnosis and treatment has led parents to seek different self-management strategies including Alternative and Complementary Medicine (CAM). The aim of this study was to perform a systematic review and meta-analysis about the use and effect of CAM modalities to treat adverse effects of conventional cancer treatment among children and young adults.

**Methods:**

Six scientific research databases were used to identify randomized controlled trials (RCTs) from 1990 to September 2020. Included studies investigated the use of CAM to treat cancer treatment related adverse effects in children and young adults compared to controls.

**Results:**

Twenty RCTs comprising 1,069 participants were included in this review. The included studies investigated acupuncture, mind–body therapies, supplements, and vitamins for chemotherapy-induced nausea and vomiting (CINV), oral mucositis, and anxiety among children and young adults who underwent conventional cancer treatment. Seven studies (315 participants) were included in the meta-analysis. The overall effect of CAM (including acupuncture and hypnosis only) on chemotherapy-induced nausea and/or vomiting and controls was statistically significant with a standard mean difference of -0.54, 95% CI [-0.77, -0.31] I^2^ = 0% (*p* < 0.00001). There was a significant difference between acupuncture and controls (*n* = 5) for intensity and/or episodes of CINV with an SMD -0.59, 95% CI [-0.85, -0.33] (*p* < 0.00001). No significant difference was found between hypnosis and controls (*n* = 2) for severity or episodes of CINV with an SMD -0.41, 95% CI [-1.09, 0.27] I^2^ = 41% (*p* = 0.19).

**Conclusion:**

Current evidence from this meta-analysis of randomized controlled trials shows that CAM, including acupuncture and hypnosis only, is effective in reducing chemotherapy-induced nausea and vomiting in children and young adults. More rigorous trials and long-term effects should be investigated if acupuncture and hypnosis are to be recommended for clinical use.

**Supplementary Information:**

The online version contains supplementary material available at 10.1186/s12906-022-03537-w.

## Background

Worldwide, approximately 400,000 children and adolescents up to 19 years old are diagnosed with cancer each year. In Norway, approximately 350 children and young adults (0–19 years) receive a cancer diagnosis yearly [1]. Cancer is among the top causes of death in children and adolescents worldwide, especially in high-income countries (HICs). The most common cancers in children are acute leukemia, brain tumors, lymphomas, bone and soft tissue sarcomas, and germ cell tumors [2]. As a result of medical advancements, survival rates for children with cancer have risen in most HICs. The increase in survival rates means that survivors have to deal with a symptom burden during and after cancer treatment [3]. Parents of children with cancer have described some of the symptoms derived from cancer treatment as pain, fatigue, emotional distress, and loss of appetite [4]. The burden brought about by conventional cancer treatments has led parents to seek different self-management strategies.

One group of self-management strategies is Complementary and Alternative Medicine (CAM). CAM is defined as a group of diverse medical health care system practices and products that are not considered part of conventional medicine [5]. If a CAM modality is used together with conventional medicine, it is considered complementary medicine. If the modality is used in place of conventional medicine, it is considered alternative medicine [6]. Although these modalities alone are not effective for anti-cancer treatment, using them complementary to conventional medicine has shown to improve the health of cancer patients [7]. Studies have reported that massage therapy [5] and acupuncture [8, 9] among others, provide benefits to patients during cancer treatment. The complementary modalities more often used among children with cancer are herbal remedies, diet and nutrition, and faith healing [10].

Although CAM use among parents of children with cancer is prevalent, studies have shown that the most common source of information on possible CAM use is friends and family [4]. In a study by Krogstad et al. [11], parents found the information from friends and family burdensome because they were unable to follow up their advice. Parents of children with cancer want accurate and reliable information on formal strategies from the healthcare providers treating their children, and from authorized sources such as the Norwegian Children’s Cancer Society [4].There is sparse research on how to cope with the adverse effects of conventional cancer treatment in children and young adults with cancer. The existing literature mostly reflects on the prevalence of the use of CAM, but it is limited to investigate the effectiveness of CAM modalities used to alleviate the symptom burden during and after conventional cancer treatment. To the best of our knowledge, this is the first systematic review of RCTs that aims to investigate CAM modalities used to cope with adverse effects of conventional cancer treatment among children and young adults. The aim of this systematic review is to review the research literature to identify any CAM modalities used to treat adverse effects of conventional cancer treatment among children and young adults and if data allows it, perform a meta-analysis to assess the beneficial effect of possible CAM modalities.

## Methods

Results were reported according to the Preferred Reporting Items for Systematic review and Meta-Analyses (PRISMA) checklist (see Supplementary file) [12].

The focus question was:

### Which CAM modalities are used to treat adverse effects of conventional anti-cancer treatment among children and young adults?

The PICOS (Population, Intervention, Comparison, Outcome and Study type) format was used when searching for relevant articles, which included the following four parts:

- **P**opulation: Children and young adults that were ever diagnosed with cancer or undergoing cancer treatment.
- **I**ntervention: Any CAM modalities.
- **C**omparison: Conventional medicine, usual care, waiting list, other CAM modalities, and placebo.
- **O**utcome: Reduction/Improvement of adverse effects (such as nausea, vomiting, toxicity, and mucositis) of conventional anti-cancer treatment.
- **S**tudy types: Single RCTs; double-blinded RCTs; cross-over RCTs, pilot RCTs and feasibility RCTs.

A protocol for the systematic review was created, submitted, and registered with PROSPERO (CRD42021216505). The protocol was registered on October 26, 2020. Six electronic databases were searched for eligible studies: AMED (EBSCO), Cinahl (EBSCO), Cochrane Central Register for Controlled Trials (Central) in the Cochrane Library (Wiley Interscience), Embase (Elsevier), PsycINFO (APA), and Medline (NLM). References of all included studies were hand-searched for additional eligible studies according to the search methodology. A manual search for gray literature was also performed using Google Scholar and books.

Search Methods: Various combinations of controlled vocabulary/thesaurus terms (eg. Mesh) and text words, adjusted for each database, were used. The following Mesh terms were used: Exp Neoplasms, exp Complementary Therapies, exp Integrative medicine, Alternative Therapies, exp Child, exp Adolescent, exp Young Adult, exp Infant, Adverse effects. sf (subheading, fs), adverse event, side effects and adverse reactions, Drug Related Side Effects and Adverse Reactions, exp Adverse drug reaction reporting systems, exp Randomized controlled trials.

These text words were used: neoplasm, leukemia, lymphoma/soft tissue sarcoma, pediatric cancer, pediatric oncology, integrative oncology, cancer treatment, childhood cancer, pediatric, palliative care, CAM modalities, CAM treatment, CAM, integrative medicine, complementary medicine, alternative medicine, unconventional medicine, spiritual healing/faith healing, children, child*,^1^ infant, adolescent, juvenile, pediatric, puberty, young adults, young person, teen*^1^, childhood, toddler, side effects, safety, risks factors, harm, adverse reactions, indirect/direct risks, adverse drug reaction, symptom management, hopelessness, suffering*.* The search string with the search terminology is attached as supplementary material.

#### Inclusion and exclusion criteria

The filters were human, Danish, Dutch, English, German, Norwegian, Spanish, and Swedish. The searches were limited to the period from January 1990 to April 2021. The inclusion comprised RCTs that reported CAM modalities to treat adverse effects of conventional cancer treatment among children and young adults. All adverse effects and CAM modalities were considered. Studies were excluded based on the following criteria: (i) studies did not report adverse effects of cancer treatment; (ii) studies unrelated to cancer or CAM; (iii) studies that were not RCTs, pilot RCTs, or feasibility RCTs; (iv) studies that were conducted among adults with cancer; (v) studies that were in languages other than the ones previously stated.

#### Study selection and data management

Search results were uploaded in the reference manager program Endnote to facilitate study selection, and a single data management file was produced identifying all references in the search process. Duplicates were removed and two authors (DCM and TS) screened the remaining references independently. Reasons for excluding articles were documented. Neither of the review authors was blind to the journal titles, study authors, or institutions. A flowchart of the study selection and identification according to the PRISMA guidelines [12] was generated.

Three authors (DCM, TS, and GO) developed the search strategy and performed the searches. The first and last authors screened the abstracts and searched for articles that met the inclusion criteria. DM and TS read the articles, extracted the data, and conducted the quality appraisal of the included articles independently. They also screened the abstracts and searched for articles that met the inclusion criteria using Rayyan web app [13].

#### Placebo

The placebo methods used consisted of sham acupuncture, sham herbs and supplements (i.e., shampoo syrup and placebo capsules), and sham products.

#### Data extraction

Data from the RCTs were extracted according to the Cochrane Handbook for Systematic Reviews of Interventions [14]. A table to extract data was created and included fifteen fields: study ID, objectives, method, design, setting, aim(s), sample size, dropout, participants (intervention/control groups), intention to treat & power calculation, inclusion/exclusion criteria, intervention (treatment vs. control), results, adverse effects due to the use of CAM, and funding.

#### Joanna Briggs Institute (JBI) quality assessment of the studies

The included trials were imported into the System for the Unified Management, Assessment and Review of Information (SUMARI—software program JBI) for methodological assessment and critical appraisal of study quality utilizing the checklist for RCTs [15]. Two authors (DM and TS) independently rated the methodological quality of the included articles using the critical appraisal checklists in SUMARI. Discrepancies between the reviewer’s quality assessments were discussed among the reviewers and resolved. Articles were scored by assigning 1 point for each yes answer and zero points for no or unclear answers. To obtain the score, the points were added, and a percentage was calculated. For this systematic review, articles with > 75% yes scores on the critical appraisal items were classified as high quality, from 50 to 74% as medium quality, and < 50% as low quality [16]. Low quality studies were excluded from further analysis.

#### Description of meta-analysis

Meta-analysis was conducted using Review Manager (RevMan) [Computer program]. Version 5.4. The Cochrane Collaboration, 2020 [17]. The study population was divided into those who received CAM modalities (acupuncture, acupressure, or hypnosis) and those who did not receive CAM for nausea and vomiting induced by conventional cancer treatment. The studies were combined into the meta-analysis if they were homogenous regarding study design, participants, intervention, control, and outcome measures. Studies that did not meet these criteria were excluded from the meta-analysis. For continuous outcomes, a random effect model was used, and standardized mean difference (SMD) with 95% confidence intervals (CI) was calculated as the difference in means between groups divided by the pooled standard deviation using Hedges’s correction for small study samples [14]. When missing standard deviations, they were calculated from standard errors, or by using the sample data provided in the article [14].

## Results

### Outcome of literature searches

**Fig. 1:**
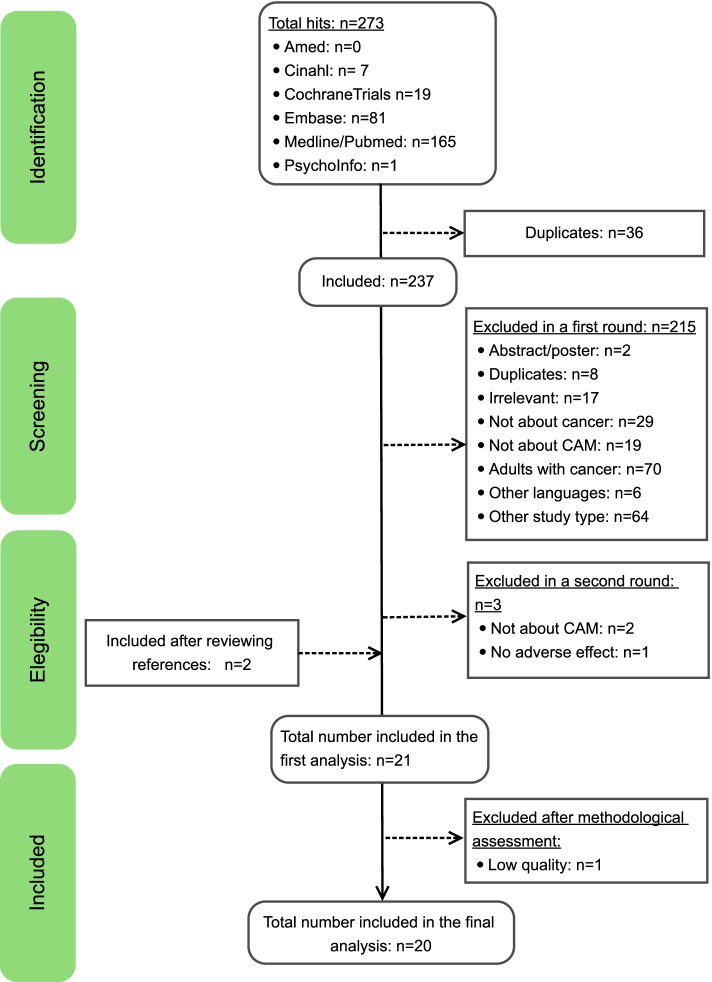
Flow chart of the inclusion process in this study

The search produced a total of 273 hits. Seven hits were identified in Cinahl, 19 in Cochrane Central Register for Controlled Trials, 81 in Embase, 165 in Medline/Pubmed, and one in Psychoinfo. After the identification process, 36 studies were identified as duplicates and therefore excluded. Studies were evaluated based on titles and abstracts. During the screening process, 215 studies were excluded for the following reasons: 2 were abstract/posters; 8 duplicates; 17 were irrelevant; 29 were not about cancer; 19 were not about CAM; 70 were about adults with cancer, and 6 were in languages other than Danish, Dutch, English, German, Norwegian, Spanish, and Swedish, 64 were other study types. In a second round, 3 trials were excluded, 2 were not about CAM, and 1 did not include adverse effects. After reviewing the references of the 19 eligible articles, the authors included 2 more RCTs that met the eligibility criteria [18, 19]. A total Twenty-one [8, 9, 18–36] RCTs comprising 1,149 participants were eligible for inclusion in this review. Among them were six [22, 23, 32, 33, 35, 37] RCTs that had included participants up to the age of 21 years. Since all these studies focused on the effectiveness of CAM in the pediatric population, the review team decided, following a discussion, to include them in the review. Upon completion of the data extraction, assessment, and critical appraisal of study quality, one [18] study was excluded because it was determined to be of low quality. Although the excluded study was included in the data extraction table, no further results were reported. Consequently, a total of 20 studies (*n* = 1,069) were included in this review (Fig. 1).

### Methodological quality of studies

Detailed characteristics of the included studies are presented in Table 1. Sample size refers to the total number of participants in the study. In the participant group, n refers to the number of participants who received the treatment or who were in the control group respectively. Dropout refers to the number of participants who left the study before completion.

**Table 1:** Characteristics of included studies

Study ID	Objectives	Method	Design	Setting	Aim (s)	Sample size	Drop-out	Participant(s) intervention/control	Intention to treat & power calculation	Inclusion/exclusion criteria	Intervention treatment vs control	Results	Adverse effects due to the use of CAM	Funding
**Alternative medical systems**														
Dupuis, L 2018 [25]	Acupressure bands for chemotherapy-induced nausea	RCT	Single-blind RCT	Medical centers in Canada	Compare CIN control in the acute phase provided by standard antiemetic agents combined with acupressure bands versus sham bands in children 4–18 yrs. of age receiving HEC***. Compare CIN^+^ control in the delayed phase compared to CIV^§^ control in the acute and delayed phases	187	22	Children 4—18 yrs. old. *n* = 83 (acupressure bands) vs 82 (sham bands). Total: (*n* = 165)	Power calculation reported	**Inclusion**: English speaking patients aged 4–18 yrs. with non relapsed cancer and with and English speaking guardian **Exclusion:** Patients with prior history of acupressure use or who planned to received antiemetic agents other than ondansetron, granisetrin, dexamethasone, or aprepitant on a scheduled basis	Acupressure bands 30 min before chemo vs. Sham band	Bands did not improve CIN or CIV control in children	Six adverse events (four in the intervention group and 2 in the sham group). Bands being too tight. No serious adverse effects reported	National cancer institute
Ghezelbash, S 2017 [30]	Acupressure for nausea-vomiting and fatigue management in acute lymphoblastic leukemia in children	RCT	Single-blind RCT	Two pediatric hospitals in Iran	Examine the effectiveness of acupressure for controlling CINV^ and CRF (cancer related fatigue)	120	0	Children ages 8–12 yrs. old. Finger acupressure *n* = 60 vs. Sham acupressure *n* = 60. Total:(*n* = 120)	NR	**Exclusion:** Patients with **l**ow platelet count (< 50,000), a bleeding disorder, hemoglobin levels < 9 g/dl and hematocrit < 30, or were on active treatment for anemia	Finger acupressure (p6, st36) vs. sham acupressure (SI3,LI12)	Significant differences were observed between the two groups based on the fatigue and nausea intensity immediately and one hour post intervention (*P* ≤ 0.001)	NR	NR
Gottschling S, 2008 [7]	Acupuncture to alleviate chemotherapy-induced nausea and vomiting	RCT	Single-blind RCT crossover trial	5 pediatric oncology centers in Germany	Evaluate the efficacy and acceptance of acupuncture as an additive antiemetic treatment during highly emetogenic chemotherapy in pediatric cancer patients	23	0	Children 4- 18 yrs. old. *n* = 23 (intervention) vs 23 (standard care only). (Participants were their own controls). Total: (*n* = 23)	Power calculation reported	**Exclusion:** Patients with full control of CINV without need of antiemetic rescue mediation during 1st chemo course. Children under 6 or over 18, previous experience with acupuncture within the last 6 months	Acupuncture vs chemotherapy/antiemetic regime	Use of rescue antiemetic medication was lower among those using acupuncture (*p* = 0.001). Episodes of vomiting among those receiving acupuncture were lower (*p* = 0.01) in pediatric oncology patients	Four cases of pain from needling. Adverse effects were minor and transient	NR
Jones, PA 2008 [32]	Acupressure for CIVN in children with cancer	Pilot study	Prospective randomized crossover clinical trial (RCT)	Children's hospital USA	Assess feasibility, safety, and effectiveness of acupressure therapy for preventing or reducing CIVN in children receiving chemotherapy	21	3	Children ages 2–20 yrs. old. *n* = 18 (acupressure band) vs 18 (placebo band). Total: (*n* = 18) (Participants were their own controls)	NR	**Exclusion:** If patients were not expected to received at least 3 courses of chemotherapy, parents did not consent, were over 7 yrs. old and did not assent to participate, no English speaking	Acupressure wrist bands vs. sham wrist bands (wrist bands placed on wrist prior to starting chemo)	Acupressure bands did not offer significant benefits	NR	NR
Reindl, TK 2006 [8]	Acupuncture for CIVN in children with cancer	RCT, multicenter crossover trial	Randomized multicenter, prospective crossover trial	4 German pediatric oncology centers	Evaluate the efficacy and acceptance of acupuncture as a supportive antiemetic approached during highly emetogenic chemotherapy	11	0	Children 6–18 yrs. old (*n* = 11). *n* = 11 (needle acupuncture) vs 11 (standard care only). Total: (*n* = 11). Participants were their own controls	Did not reach power	**Inclusion:** Children who received several courses of highly melogenic chemotherapy as part of therapy protocols for Ewing's sarcoma, rhabdomyosarcoma, and osteosarcoma, including 5-HT3 antagonists as basic antiemetic medication	Antiemetic medication vs. Antiemetic medication plus acupuncture. Acupuncture was applied on day 1 and throughout the chemotherapy course	Acupuncture enable patients to experience higher levels of alertness during chemo and reduced nausea and vomiting	One case of needle pain	CD Foundation and Friedrich-Sicker Foundation
Varejão, C 2019 [29]	Laser acupuncture for relieving nausea and vomiting in pediatric patients undergoing chemotherapy	Single-blinded RCT	Single-blind randomized clinical trial	Oncology Hospital in Rio de Janeiro, Brazil	1.Apply laser acupuncture in children and adolescents undergoing chemo 2. Analyze the effects of laser acupuncture in terms of preventing and/or relieving nausea and vomiting 3. Propose a nursing care protocol using laser acupuncture to prevent and/or relieve nausea and vomiting	18	1	Children between 6–17 yrs. old. *n* = 7 (intervention) vs 10 (sham acupuncture). Total:( *n* = 17)	Power calculation reported and reached	**Inclusion:** Children 6–17 yrs. with solid tumors. Patients going chemo with cisplatin, methotrexate, doxorubicin, etoposide, infosfamide, and/or cyclophosphamide. Use of medium and/or highly emetogenic drugs **Exclusion:** previous history of gastrointestinal diseases or antiulcer treatment. Prior history of acupuncture treatment. Use of aprepitant	Laser acupuncture vs sham laser acupuncture. A total of 26 treatments in each group ( 1 day of chemo)	Significant relief from nausea in the intervention group ( *p* < .0005). Decrease in the number of vomiting episodes on the 2nd and 3rd day of chemotherapy (*p* = .0001)	NR	Provided by researchers. No funding from companies or public agencies
Yeh, CH 2012 [18]	Auricular acupuncture for nausea and vomiting	Pilot study crossover trial	Randomized single blinded crossover trial	Hospital in Taiwan	Determine if auricular acupressure point is more effective than sham acupressure point and standard care for chemotherapy induced nausea and vomiting	17	7	Children ages 5–18 yrs. old *n* = 10 auricular acupressure, *n* = 10 Sham acupressure intervention. Total: (*n* = 10). Participants were their own controls	Power calculation reported	**Inclusion:** Children diagnosed with cancer and that had at least one round of chemotherapy treatment who were prescribed chemotherapy drugs with high or moderate level of emetogenic agents; were prescribed standard antiemetics with their chemotherapy drugs and had not previously received any acupuncture or acupressure treatments in the previous 3 months	Auricular acupressure (AAP) vs. Sham acupressure intervention (SAP)	Patients in the auricular acupuncture point group had lower occurrence and severity of acute and delayed nausea (*p* = 0.0289) and shorter vomiting (*p* = .0024) duration than patients receiving sham acupuncture and standard care	NR	NR
**Biological-based therapies**														
Consolo, Lzz 2013 [24]	Zinc supplement for weight gain and infectious episodes in children with acute leukemia	Double blinded RCT	Double blind placebo controlled study	Regional Hospital in Brazil	Evaluate the effects of oral zinc supplementation on weight gain and infectious episodes in children and adolescents with acute leukemia	38	0	Children 1–18 yrs. old, *n* = 20 (intervention, syrup with zinc) vs *n* = 18 (placebo, only syrup). Total (*n* = 38)	NR	**Inclusion:** Children with previously established clinical and laboratorial diagnoses either for lymphocytic or myeloid leukemia. **Exclusion:** acute infectious disease, renal failure, post surgery status or usage of zinc containing drug	Group A: Oral placebo syrup containing no zinc. Group B syrup containing zinc	Significant difference was found in favor of the intervention group regarding weight gain (*p* = 0.032) and the number of infections episodes ( *P* = 0.02). No significant differences in xerostomy, taste dysfunction nausea and vomiting between the two groups (*P* = 0.812)	NR	Brazilian agencies CNPq and FUNDECT-MS
El-Housseiny, AA 2007* [19]	Effectiveness of vitamin E to treat mucositis	RCT	Randomized controlled trial	Oncology department Alexandria University and El-Talaba hospital of Alexandria	To compare the effect of vitamin E topically and systematically in the treatment of chemotherapy induced oral mucositis	80	17	Children under 12 yrs. old *n* = 30 (Vitamin E topical), *n* = 33 (Vitamin E systemic).Total: (*n* = 63)	NR	**Inclusion**: Children with chemotherapy induced oral mucositis	Topical Vitamin E application vs. Systemic Vitamin E intake	Topical application of Vitamin E twice daily was significant more effective than systemic Vitamin E for chemotherapy-induced mucosis ( *P* > 0.001)	NR	NR
Evans, A 2018 [26]	The use of aromatherapy to reduce CIN in children with cancer	Double-blinded RCT, with three arms	double blind placebo controlled study	Infusion center for emetogenic chemo in So. California	To investigate the utility of ginger aromatherapy in relieving chemotherapy-induced nausea in children with cancer	49	0	Children 8 to 21 yrs. old, *n* = 10 (water gr), *n* = 19 (shampoo gr), *n* = 20 (ginger gr). Total: (*n* = 49)	Power calculation /Intent to treat reported	**Inclusion:** Diagnoses of cancer with any type and amount of prior therapy. Thirty minute infusion of moderately emetogenic chemotherapy. **Exclusion:** Patients with asthma on daily medication. Patients unable to complete the four point face scale	Aromatherapy (inhalation of ginger aroma oil) vs. no treatment (inhalation of water) vs. placebo (shampoo)	Ginger aromatherapy did not significantly decrease nausea. Fifty-nine percent (*n* = 29) reported no change while 29% (*n* = 14) reported improvement	NR	J. Patrick Barnes Grant from the DAISY foundation
Khurana, H 2013 [27]	An evaluation of Vitamin E and Pycnogenol (P) in children suffering from oral mucositis during cancer chemotherapy treatment	Single blinded RCT	Single-blind randomized controlled clinical trial	CSM Medical University, Lucknow. India	Evaluate P for its beneficial effects on oral mucositis in children and to compare with E	72	0	Children 16–15 yrs. old *n* = 24 (Pycnogenol); *n* = 24 (Vitamin E); *n* = 24 (Placebo (glycerine)). Total: (*n* = 72)	NR	**Inclusion**: Children receiving chemotherapeutic regime with signs of chemotherapy-induced mucositis and patients whose parent /guardian provided consent. **Exclusion:** Children who received chemotherapy in the head and neck region, on anti-platelet or anticoagulant therapy, having pre-existing oral disease	Preparations of Vitamin E vs Pycnogenol vs Glycerine	Both drugs Vitamin E and Pycnogenol were effective for treatment of oral mucositis compared to placebo (*P* < 0.001)	NR	NR
Ladas, EJ 2010 [22]	Milk thistle (Silybum marianum) for the treatment of hepatotoxicity in childhood ALL^^	A double-blind RCT	Randomized, controlled, double-blind study	Columbia University Medical Center	To evaluate the safety and feasibility of Milk thistle for the treatment of hepatotoxicity in children with ALL who are receiving maintenance-phase chemotherapy	50	1	Children between 1 -21 yrs. old, *n* = 23 (Milk Thistle), *n* = 26 (placebo). Total: (*n* = 49)	Power calculation reported	**Inclusion:** Children with ALL. Maintenance phase of therapy Hepatic toxicity of grade 2 or greater on ALT, AST, total bilirubin. **Exclusion**: Patients with extra hepatic biliary obstruction, or malabsorption syndromes	Milk thistle vs placebo for 28 days	Milk Thistle was associated with trend toward significant reductions in liver toxicity (AST* *P* = .05; ALT** *P* = .07)	Seven cases of adverse effects in the intervention group: Diarrhea (*n* = 2), flatulence (*n* = 1),irritability (*n* = 2) and stomach ache (*n* = 2). Six cases in the placebo group: Decreased appetite (*n* = 1), Diarrhea (*n* = 2), Stomach ache (*n* = 2), soft stools (*n* = 1). No significant differences in patient reported adverse effects	American Institute for Cancer Research. The Tamarind Foundation. Part of NCI grant
Pillai AK, 2011 [33]	Ginger powder vs. Placebo as an add-on therapy in children and young adults receiving high emetogenic chemotherapy	Double-blind RCT	Prospective double-blind, randomized single institutional study	All India Institute of medical sciences,New Delhi, India	To evaluate the efficacy of ginger powder in reducing CINV	60	3	Children and young adults 8–21 yrs. old, *n* = 30 (Ginger gr), *n* = 27 (Placebo gr),Total: (*n* = 57)	NR	**Inclusion:** Children newly diagnosed bone sarcomas undergoing therapies with high emetogenic chemo. **Exclusion:** Children with weight < 20 kg or > 60 kg, those receiving radiotherapy and patients additionally receiving aprepitnat with the standard antiemetics were excluded	Ginger root powder capsules vs placebo	Ginger root powder significantly reduced the severity of both acute and delayed CINV (*p* = 0.003); Acute vomiting (*p* = 0.002); Delayed nausea (*p* = < 0.001); Delayed vomiting (*p* = 0.022)	NR	NR
Rathe, M 2019 [21]	Bovine colostrum against chemotherapy-induced gastrointestinal toxicity in children with ALL	Double-blinded RCT	Double blind placebo controlled clinical trial	Hans Christian Andersen Children's Hospital, Odense University Hospital and Rigshopitalet. University Hospital of Copenhagen, Denmark	To investigate nutrition supplementation with bovine colostrum effect on fever, infectious morbidity, and mucosal toxicity during induction treatment for childhood ALL	62	0	Children 1–18 yrs. old, *n* = 30 (treatment), *n* = 32 (placebo), Total: (*n* = 62)	Power calculation and intention to treat analysis performed	**Inclusion:** Newly diagnosed with ALL. **Exclusion**: Children with known lactose intolerance or allergy to cow's milk protein	Received daily dietary supplement with either bovine colostrum or a placebo supplement from the first day of chemotherapy until day 29 or end of induction therapy	Peak severity of oral mucositis was significantly reduced by colostrum compared with placebo (*p* = 0.02). No difference was observed for days of fever, neutropenic fever, intravenous antibiotics, or incidence of bacteremia	No adverse effects of the supplement were reported	Danish Childhood Cancer Foundation, Odense University Hospital research fund, common research fund b/w Odense University Hospital and Rigshospitalet and University of Southern Denmark
Tomaževič T, 2013 [23]	Propolis (bee glue) for effectiveness in the treatment of severe oral mucositis in chemotherapy treated children	Single blinded RCT	Single-blind randomized controlled clinical trial	Slovenia University children's hospital	Assess the efficacy of propolis versus placebo for the treatment of chemotherapy induced oral mucositis	50	10	Children 1–19 yrs. old (*n* = 19 propolis) vs (*n* = 21, placebo). Total:( *n* = 40)	Power calculation reported	**Inclusion:** Pediatric patients who had been diagnosed with cancer and had started chemotherapy. **Exclusion**: Allergy to propopolis and pre-diagnosed oral disease or therapy for oral disease	Propolis vs placebo	No significant difference were found between the groups.Propolis cannot be recommended for severe oral mucositis	No adverse effects of the supplement were reported	Colgate Palmolive Adria
Wada M, 2010 [34]	Effects of the administration of Bifidobacterium breve (probiotic) on patients undergoing chemotherapy for pediatric malignancies	Single-blinded RCT	Single-blinded, placebo controlled trial	Juntendo University Hospital, Tokyo Japan	To evaluate the effects of probiotic, Bifidobacterium breve, and its ability to prevent infection, fecal micro flora, and intestinal environments in cancer patients on chemotherapy	40	2	Children ages 1–13 yrs. old, *n* = 17 (probiotic), *n* = 21 (placebo), total: (*n* = 38)	Power calculation reported	**Exclusion**: Presence of congenital immune deficiency and oral intake of the probiotic during 2 weeks prior to the trial	Probiotic vs. placebo	Frequency of fever (*p* = 0.02) and use of IV antibiotics were lower in the probiotic group (*p* = 0.04), suggesting that probiotic could be beneficial for immunocompromised hosts by improving intestinal environment	NR	NR
Ward E, 2009 [35]	The effect of high-dose enteral glutamine on the incidence and severity of mucositis in pediatric oncology patients	RCT cross-over trial	Randomized controlled	St. James's University Hospital, Leeks, UK Yorkshire Regional Center for Pediatric oncology	To determine if enteral glutamine daily for 7 days was effective in reducing the incidence an severity of mucositis in pediatric oncology patients	76	26	Children between 1–21 yrs., total: (*n* = 50). Patients were their own control	Power calculation reported	**Inclusion**: Patients who had two identical courses of chemo and were receiving chemotherapy likely to cause mucositis	Glutamine vs. placebo administered daily for 7 days	The study showed that high-dose enteral glutamine did not reduced the incidence and severity of oral mucositis as determined by subjective toxicity measurements, but did show a significant reduction in parenteral nutrition usage (*p* = .049)	NR	SHS International (provided glutamine)
**Mind–body therapies**														
Abdulah, DM 2018 [31]	Investigated group art therapy on quality of life in pediatric patients with cancer	RCT	Randomized controlled trial	Heevi Pediatric Hospital in Duhok, Iraq	To evaluate the effectiveness of art therapy on the health related quality of life for children undergoing chemotherapy	61	1	Children ages 7–16 yrs. old, *n* = 30 (treatment), *n* = 30 ( control). Total: (*n* = 60)	Power calculation reported	**Inclusion:** Children previously diagnosed with cancer and had received chemotherapy for at least the last 6 months. **Exclusion:** Patients who attended fewer than six two hours painting and drawing sessions	Art therapy ( painting) vs control ( not explained)	Patients in the experimental group were more physically active and energetic (*p* < 0.001), were less depressed and had fewer stressful feelings (*P* = 0.004). They also had more opportunities to structure and enjoyed their social and leisure time and participation in social activities (*P* = 0.003), creates more social relationships (*P* = 0.043) and had better overall health (*P* < 0.001)	NR	NR
Jacknow, DS 1994 [20]	Hypnosis in the prevention of CINV in children	Single-blinded RCT	Randomized and controlled single-blind trial	Lucille Salter Packard Children's Hospital (Stanford Univ.) and Moffitt/Long Hospitals (Univ. Of California- San Francisco)	To study the effectiveness of hypnosis for decreasing antiemetic medication usage and treatment of CINV	20	1 loss to follow-up, data was used in the analysis	Children ages 6–18 yrs. old, *n* = 10 (treatment), *n* = 10 (control), Total: (*n* = 20)	NR	**Inclusion**: Newly diagnosed patients with no previous experience with chemotherapy. **Exclusion:** Evidence of central nervous system disease	Hypnosis and antiemetics vs standard anti-emetic regimen/conversation with therapist	The hypnosis group experienced less anticipatory nausea than the control group (*p* < .02) and used less antiemetic medication (*p* < .04)	NR	DHHS Maternal and child health bureau
Nguyen, TN 2010 [28]	Music therapy to reduce pain and anxiety in children with cancer undergoing lumbar puncture (LP)	Single-blinded RCT	Single blinded randomized clinical trial	National Hospital of pediatrics. Hanoi. Vietnam	To evaluate if music therapy influences pain and anxiety in children with cancer	40	0	Children 7–12 yrs. old, *n* = 20 (treatment), *n* = 20 (control). Total: (*n* = 40)	Power calculation reported	**Inclusion:** Patients that were due to undergo LP and had previously undergone LP at least once previously **Exclusion:** Children had any significant hearing or visual impairments or cognitive disorder	Music vs control (no music)	Lower pain (*p* < .003), respiratory rate (*p* < .003) and anxiety scores (*p* < .001) were significant lower in music group after the LP compared with the control group	NR	There was no financial support
Zeltzer, LK 1991 [36]	Behavioral intervention (hypnosis) for chemotherapy distress in children	RCT	Randomized controlled trial	Two pediatric oncology centers University of Texas Health Science Center in San Antonio and Children's Hospital of Los Angeles	To determine the relative efficacy of the two forms of behavioral intervention for reducing chemotherapy related distress	54	0	Children 5–17 yrs. old, *n* = 21 (hypnosis), *n* = 16 (support), *n* = 17 (control), Total: (*n* = 54)	Not reported	**Inclusion:** Children with cancer, reliable reporting of significant chemotherapy related nausea and /or vomiting during baseline assessment and were able to consistent independent self reports of their chemotherapy related distress. **Exclusion:** Patients that could not provide reliable consistent reporting	Hypnosis vs support (relaxation) vs control ( causal conversation)	Children in hypnosis group reported the greatest reduction of both vomiting (*p* = < .005) and shorter duration of nausea (*p* = < .001)	NR	Grant from the National Cancer Institute

^^ ALL: Acute lymphoblastic leukemia *AST: Aspartate amino transferase**ALT: Amino alanine transferase***HEC: Highly melogenic chemotherapy: §CIV:chemotherapy induced vomiting: + CIN:chemotherapy induced nausea:CINV^: chemotherapy induced nausea-vomiting: NR: Not reported in publication *excluded from further analysis due to low quality

Seven [18–23] of the 21 studies did not report sources of funding, and two studies [24, 25] stated that they received no financial support. Eight [18–21, 24, 26–28] studies did not report power calculations.

Fifteen studies (*n* = 15*)* [8, 9, 19–21, 24, 26, 27, 29–35] were assessed as high quality because they had scores of 75% or higher (Table 2). Two studies (*n* = 2) [29, 30] met the criteria for 13 out of 13 items (see Table 2). Seven studies (*n* = 7) [20, 25, 26, 31–33, 35] addressed 12 items, and six studies (*n* = 6) [8, 9, 19, 21, 24, 27] addressed ten items. Five studies (*n* = 5) [22, 23, 28, 36, 37] were assessed as medium quality because they obtained scores between 50 and 74%. Two studies (*n* = 2) [22, 36] addressed nine items, and three studies (*n* = 3) [23, 28, 37] addressed eight items. One (*n* = 3) [18] paper was assessed as low-quality (< 50%) as it addressed only 5 out of 13 items, and was excluded from further analysis.

**Table 2:**
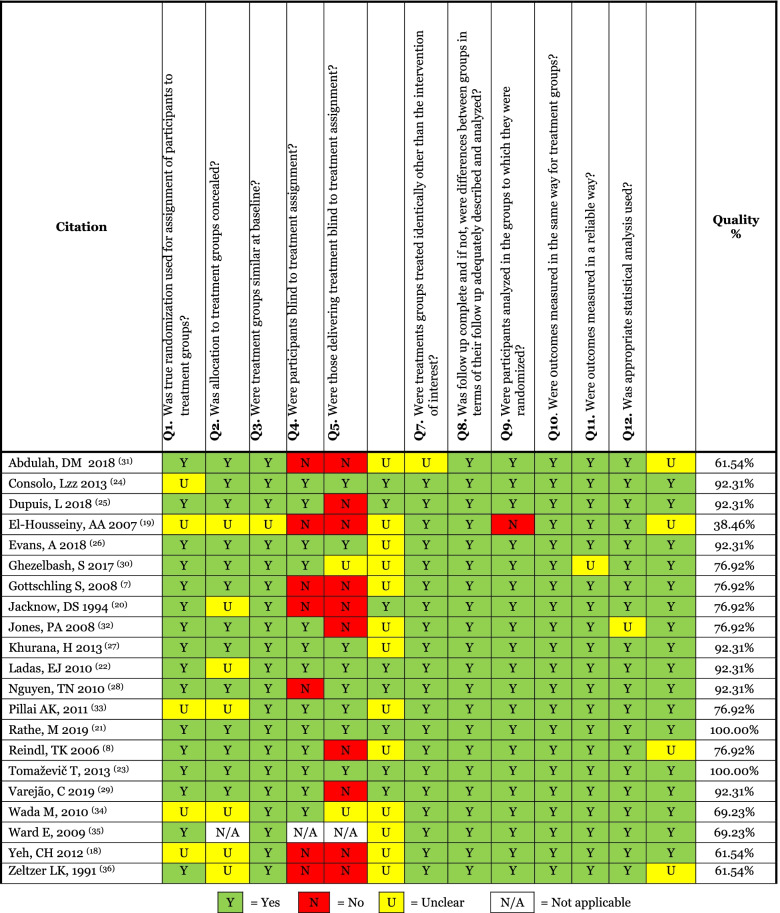
Studies quality assessment

< 50% = Low-quality 50% -75% = Medium-quality > 75% = High-quality

### CAM modalities

The results of the literature search indicate that the existing RCTs about the use of CAM modalities to alleviate the adverse effects of conventional cancer treatment in children and young adults can be divided into three main areas: Alternative medical systems, biological-based therapies, and mind–body therapies. The search returned seven [8, 9, 19, 24, 31, 35, 37] RCTs that have been conducted using acupuncture as a treatment for chemotherapy-induced nausea and vomiting. Ten [18, 20–22, 26, 29, 30, 33, 36, 38] studies emerged where supplements such as zinc, vitamin E, aromatherapy, pycnogenol, milk thistle, ginger powder, bovine colostrum, propolis, glutamine, and probiotics were examined in the treatment of adverse effects such as oral mucositis, nausea, vomiting, hepatotoxicity, fever, and the prevention of infection. Lastly, four [23, 25, 27, 28] studies emerged where mind–body therapies were used to treat stress, anxiety, nausea, vomiting, and to improve the quality of life among children and young adults with cancer undergoing treatment.

#### Alternative medical systems

All of the studies related to alternative medical systems investigated if different acupuncture treatments could alleviate chemotherapy-induced nausea and vomiting among children and young adults undergoing conventional cancer treatment. Acupressure was used in four of the studies, two [19, 31] used wristbands, one [37] used auricular seeds, and one [24] used fingers. Two studies [8, 9] used needle acupuncture and one [35] used laser acupuncture. Neither of the studies accessing treatment with wristbands [19, 31] showed any significant difference in nausea and vomiting between the intervention and control groups (sham acupuncture, standard care). Although insignificant, Yeh et al. [37] found that patients receiving seed auricular acupuncture had lower occurrence of acute and delayed nausea and shorter vomiting duration than patients receiving sham acupuncture and standard care. Ghezelbash et al. [24] found a significant difference in lower nausea intensity in the intervention and placebo groups immediately (*p* = 0.02) after and one hour (*p* ≤ 0.001) after intervention. The fatigue intensity was also considerably reduced in both groups one-hour post-intervention (*p* ≤ 0.01). Gottschling, et al. [8] found that the need for rescue antiemetic medication was significantly (*p* < 0.001) lower during acupuncture courses compared to control courses, and episodes of vomiting per course were significantly lower in courses with acupuncture (*p* = 0.01). Reindl et al. [9] found that antiemetic medication used was reduced in courses with acupuncture (*p* = 0.024) compared to the courses where acupuncture was not used. Vereajão et al. [35] found that laser acupuncture relieved nausea during chemotherapy (*p* < 0.0001) and relieved vomiting on the second and third day after chemotherapy (*p* = 0.0001) compared to those receiving sham laser acupuncture.

In conclusion, two [8, 9] studies found that acupuncture treatment lowered the use of antiemetic medication. Also, two [8, 35] studies found that acupuncture relieved vomiting during treatment, and one [35] study found that it relieved nausea post-chemotherapy treatment, however, at an insignificant level (for further information see the meta-analysis section).

#### Biological-based therapies

Ten studies [18, 20–22, 26, 29, 30, 33, 36, 38] identified in the literature search evaluated the effectiveness of supplements, such as vitamin E, zinc, ginger, bovine colostrum, propolis, probiotics, and glutamine, on alleviating chemotherapy-induced adverse effects such as oral mucositis, nausea, vomiting, hepatotoxicity, weight loss, and infection. The use of ginger aromatherapy to treat nausea and propolis to treat oral mucositis showed insignificant difference between the intervention and control groups [30, 38]. Consolo et al. [26] found that children taking zinc had significant (*p* = 0.03) weight gain and fewer infections (*p* = 0.02) compared to those in the control group. Three studies showed a significant effect of CAM modalities on oral mucositis. Khurana et al. [20] evaluated the effects of vitamin E and pycnogenol among children suffering from oral mucositis during cancer chemotherapy. Results showed significant improvements in mucositis among those who received vitamin E and pycnogenol treatment (*p* < 0.001) compared to those in the control group. Ward et al. [36] investigated the effect of enteral glutamine on the incidence and severity of mucositis among children and young adult oncology patients. Glutamine did not reduce the severity or incidence of mucositis, but the use of parenteral nutrition was significantly reduced (*p* = 0.049). Rathe et al. [29] evaluated the efficacy of bovine colostrum to treat chemotherapy-induced gastrointestinal toxicity, the incidence of fever, and infectious complications among children with cancer. The results showed no difference between the experimental and control groups among gastrointestinal toxicity and incidence of fever but there was a significant (*p* = 0.02) reduction in the severity of oral mucositis among participants who received bovine colostrum when compared to those in the control group [29].

Ladas et al. [33] looked at the effectiveness of using milk thistle for the treatment of hepatotoxicity. Milk thistle did not show any significant difference in frequency of adverse effects, incidence or severity of toxicity, or infections. Participants receiving milk thistle treatment did, however have significantly (*p* = 0.05) lower aspartate aminotransferase (AST) measurements on day 28 and 56. Pillai et al. [21] investigated the effectiveness of ginger powder in chemotherapy-induced nausea and vomiting. The findings showed that acute moderate to severe nausea (*p* = 0.003) and vomiting (*p* = 0.002), and delayed moderate to severe nausea (*p* < 0.001) and vomiting (*p* = 0.02) were significantly more common among the control group participants compared to those in the experimental group. Lastly, Wada et al. [22] evaluated the effects of probiotic *bifidobacterium breve* among children undergoing chemotherapy. Results showed that the frequency of fever (*p* = 0.02) and the use of intravenous antibiotics (*p* = 0.04) were significantly lower in the participants receiving probiotics than those in the placebo group.

In summary, several biological-based therapies have been shown to have positive effects on children and young adults undergoing anti-cancer treatment. Zinc helped children gain weight and had fewer infections [26]. The severity of mucositis was reduced among those who took vitamin E, pycnogenol, and bovine colostrum [20, 29]. Glutamine decreased the use of parenteral nutrition [36]. Milk thistle lowered the AST measurements [33]. Probiotic *bifidobacterium breve* lowered the frequency of fever and the use of intravenous antibiotics [22]. Lastly, ginger powder reduced acute and delayed nausea/vomiting [21].

#### Mind–body therapies

Four studies (*n* = 4) [23, 25, 27, 28] assessed the use of mind–body therapies such as hypnosis, music and art therapy to treat chemotherapy-induced adverse effects (i.e., nausea, vomiting, stress, anxiety, and pain). Two of the studies (*n* = 2) [27, 28] evaluated the use of hypnotherapy to treat chemotherapy-induced nausea and vomiting. Jacknow et al. [27] found that patients receiving hypnosis treatment used less supplemental antiemetic medication compared to those in the control group during the first (*p* < 0.04) and second (*p* < 0.02) course of chemotherapy. The research group also found that participants receiving hypnosis treatment experienced less anticipatory nausea (*p* < 0.02) than those in the control group [27]. In a different study, Zeltzer et al. [28] examined the effects of hypnosis and support groups on reducing chemotherapy-related distress. They found that the duration of nausea was significantly shorter for those in the hypnosis (*p* < 0.001) and support (*p* < 0.01) groups compared to those in the control group. Shorter duration of vomiting was also significant among the patients in the hypnosis group compared to those in the control group (*p* < 0.005) [28]. Music therapy was used as a treatment to reduce pain and anxiety in children with cancer undergoing lumbar puncture. Nguyen et al. [25] found that those receiving music therapy during and after lumbar puncture had significantly lower pain scores during (*p* < 0.001) and after (*p* < 0.003) the procedure. Anxiety scores were also lower among those receiving music therapy (*p* < 0.001). There was a significant reduction in respiratory rate (*p* = 0.009) and heart rate (*p* = 0.009) in children receiving music therapy during the procedure. There were also significant differences in respiratory rate (*p* = 0.003) for the children in the music group after the procedure [25]. Abdulah et al. [23] measured the effectiveness of group art therapy on the quality of life in pediatric patients. They found that those in the art therapy group were significantly more physically active (*p* < 0.001), less depressed, less emotional, and less stressed (*p* = 0.004). The results also showed that they enjoyed their leisure time more and participated in more social activities (*p* = 0.003). They also showed improvement in their relationships with other children (*p* = 0.043) and had better overall health status (*p* < 0.001) [23].

In conclusion, mind–body therapies have shown to have positive outcomes on the adverse effects experienced by children with cancer undergoing treatment. Hypnosis decreased the need for supplemental antiemetic medication and reduced anticipatory nausea [27] and the duration of nausea/vomiting [28]. Music therapy decreased anxiety and pain as well as respiratory and heart rate during treatment procedures, and also decreased the respiratory rate after treatment [34]. Finally, art therapy had a positive impact on the quality of life of the children undergoing cancer treatment [23].

### Safety of CAM interventions

Six studies (*n* = 6) [8, 9, 29–31, 33] reported adverse effects from the interventions. Among the acupuncture studies, three (*n* = 3) [8, 9, 31] reported adverse effects. Dupuis et al. [31] reported six (*n* = 6) adverse effects of bands being too tight. Gottschling et al. [8] reported four (*n* = 4) cases of pain from needling, and Reindl et al. [9] reported one case of needle pain. Among the biological-based therapies, Ladas et al. [33] reported seven cases of adverse effects as follows: diarrhea (*n* = 2), flatulence (*n* = 1), irritability (*n* = 2), and stomachache (*n* = 2). Rathe et al. [29] and Tomaževič et al. [30] noted no adverse effects reported by the participants in their RCTs.

In conclusion, only twenty-nine percent (*n* = 6) of the RCTs collected data on safety. Adverse effects were reported as mild and transient, suggesting that the therapies presented in this review have minor risks. No cases of serious adverse effects were reported.

### Meta-analysis on nausea and vomiting

**Fig. 2:**
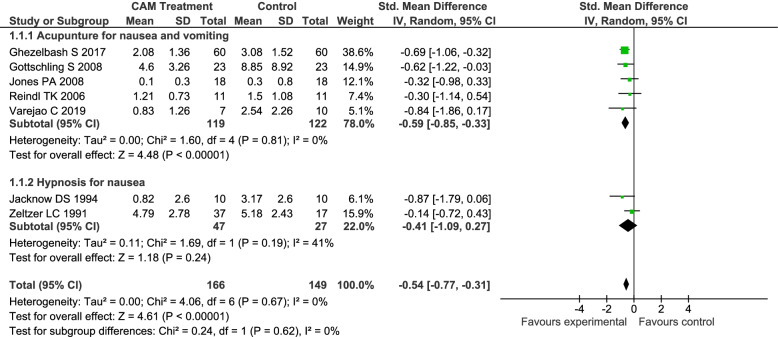
Forest Plot CAM Treatment vs. Control for CINV

Seven randomized control trials (*n* = 7) [8, 9, 19, 24, 27, 28, 35] with 315 participants were included in the statistical analysis. Studies in the meta-analysis consisted of two group interventions (*n* = 166) (acupuncture and hypnosis) versus control (*n* = 149) (standard medical care and placebo) (Fig. 2). Conventional standard care consisted of standard antiemetic medicines.

#### Overall effect of CAM for CINV

An overall comparison was made between CAM modalities (included acupuncture and hypnosis only) for chemotherapy-induced nausea and/or vomiting and controls. The difference between participants treated with CAM those in the control group was statistically significant with a standard mean difference of -0.54, 95% CI [-0.77, -0.31] I^2^ = 0% (*p* < 0.00001). The participants that received CAM treatment reported less episodes and intensity of nausea and/or vomiting.

Different sensitivity analyses were performed according to the categories of CAM treatment and are presented below. All studies eligible for the meta-analysis, with the exception of one [19], were performed among children aged 18 years or younger.

#### Acupuncture for nausea and/or vomiting

A comparison was made between acupuncture treatments for chemotherapy-induced nausea and/or vomiting and controls. Five studies (*n* = 5) [8, 9, 19, 24, 35] with 241 participants (intervention *n* = 119, control *n* = 122) were included in this comparison. A statistically significant difference was found between those who received acupuncture and those who did not -0.59, 95% CI [-0.85, -0.33] I^2^ = 0% (*p* < 0.00001) (Fig. 2). The participants that received acupuncture treatment reported less episodes and/or intensity of nausea and/or vomiting during or at the end of chemotherapy treatment.

#### Hypnosis for nausea and/or vomiting

A comparison was made between hypnosis treatments for chemotherapy-induced nausea and/or vomiting and controls. Two studies (*n* = 2) [27, 28] with 74 participants were included in this comparison (intervention *n* = 47, control *n* = 27). No statistically significant difference was found between those who received hypnosis and those who did not -0.41, 95% CI [-1.09, 0.27] I^2^ = 41% (*p* = 0.19) (Fig. 2).

We excluded 13 studies from the meta-analysis due to the following reasons:

1. The studies (*n* = 13) [20–23, 25, 26, 29–33, 36, 37] presented incomparable outcomes and CAM treatments
2. The reported data was inadequate to conduct a meta-analysis in four studies (*n* = 4) [20, 21, 29, 38]

## Discussion

This systematic review and meta-analysis demonstrate that CAM may be beneficial in relieving adverse effects of cancer treatment among children and young adults. Twenty RCTs comprising 1069 participants were included in this review. The majority (62%) of the included studies were assessed to have high methodological quality according to the JBI SUMARI tool. CAM modalities used for treating adverse effects of cancer treatment were: aleternative medical systems, biological-based therapies, and mind–body therapies. According to this review, CAM modalities helped relieve nausea, vomiting, mucositis, weight loss, anxiety, pain, and improve the overall quality of life measures. The meta-analysis demonstrated that acupuncture was effective in relieving chemotherapy-induced nausea and vomiting compared to controls.

### Alternative medical systems

Acupuncture is a promising modality for treating chemotherapy-induced nausea and vomiting in children and young adults with cancer. The results of this review are in line with other studies showing that acupuncture is beneficial. It is also included in the guidelines to treat nausea and vomiting in cancer care among both adults [39, 40] and children [41]. Acupuncture is considered to be a modality that is less invasive, more natural, and less liable to adverse effects than many conventional forms of treatment, [42] and potentially cost-effective [43]. Studies conducted among adults have demonstrated that acupuncture is effective for the management of nausea and vomiting. However, studies conducted among children are few and tend to have small sample sizes [8, 9, 19, 35, 44]. The results of this review are important because all studies included in the meta-analysis were assessed as high-quality RCTs and demonstrated a statistically significant effect towards acupuncture to treat chemotherapy-induced nausea and vomiting. The results from the meta-analysis show that overall CAM (-0.54, 95% CI [-0.77, -0.31]) (included acupuncture and hypnosis only) and acupuncture (-0.59, 95% CI [-0.85, -0.33]) have a medium effect size as defined by Cohen, J (1988) [45]. There are no existing comparison studies to establish the clinical significance of the results. However, when compared to the effect sizes of conventional emetic treatments, most of them have small or medium effect sizes [46–50]. Although emetic treatments and acupuncture are not comparable, the results from this review suggest that the use of acupuncture as a complement to conventional emetic treatment might be beneficial for the patients to control CINV.

### Biological-based therapies

Biological-based therapies including herbs and vitamins are among the most frequently used CAM modalities by children with cancer [10]. Similar to previous studies reviewing CAM use among pediatric oncology patients [5], we found that biological-based therapies were the most commonly researched modalities used for chemotherapy-induced symptoms among children and young adult oncology patients. Ten of the twenty-one studies included in this review were related to biologically based products such as vitamin E, zinc, ginger, and bovine colostrum. This is in line with Bishop et al. [51] who reported in a systematic review that the most commonly used modalities were herbal remedies, diets, and nutrition. Seven studies were classified as high quality [20, 21, 26, 29, 30, 32, 33], two as medium quality [22, 36], and one as poor quality [18]. Given the different outcomes and treatment modalities investigated, it was not possible to perform a meta-analysis with these studies. The high prevalence in the use of biological-based therapies among children and young adults with cancer indicates that further research should be conducted to further assess the existing modalities being used and others that have not yet been properly researched [52]. Some supplements have known interactions with chemotherapy [53, 54]. In the studies included in this review, there were no major drug interactions or adverse effects reported. One study [33] reported some minor adverse effects with the use of milk thistle, and two [29] reported no adverse effects.

### Mind–body therapies

Psychosocial factors play a significant role in disease onset and progression, and people’s quality of life. Hence, mind–body therapies play an important role in mitigating and controlling symptoms derived from cancer treatment [55]. Several studies have investigated the effectiveness of mind–body therapies on the treatment of anti-cancer treatment-induced symptoms and quality of life [56]. Four of the studies included in this review were related to mind–body therapies and classified as medium (*n* = 3) or high-quality (*n* = 1) studies. Two of the studies related to hypnosis were included in the meta-analysis and the overall effect on nausea and pain was insignificant. However, previous research [57] reported that hypnotherapy significantly reduced cancer-related procedural pain combined with standard care (*p* < 0.00001). Despite insignificant effect, it should be noted that the current meta-analysis was conducted with only two studies. Therefore, more randomized controlled trials should be conducted to have a larger sample size and improve the estimated effect of hypnosis on CINV. Studies excluded from the meta-analysis showed a significant effect of music and art therapy on the quality of life, and relief of symptoms such as pain and anxiety among children undergoing cancer treatment. These results are in line with other studies [58] which confirm that music and art therapy have positive effects on symptoms of anxiety and pain among children.

After reviewing the literature, it is noticeable that there is a great mismatch between the vast number of papers describing the prevalence of CAM use among children with cancer and the studies researching the effect of those treatments. The lack of RCTs in this field might be because it is more challenging (parents do not want to add extra burden to the child, risk of liability, etc.) to conduct trials in children than in adults, especially concerning cancer. Therefore, the number of studies as well as the number of pediatric patients in studies are still limited [59, 60]. The lack of studies can also be due to lack of funding to conduct CAM research [61].

This systematic review must be interpreted in light of its limitations. We may have overlooked some studies even though we carefully searched the literature in several databases and the gray literature. Also, limiting the studies to English, German, Dutch, Spanish, and the Scandinavian languages might have led us to miss relevant papers. Including pilot and feasibility studies might also be considered a limitation. However, it was important to include these studies due to limited body of work to investigate the effects of CAM modalities to treat the adverse effects of cancer treatment among children and young adults. Another limitation is that it included six articles where the age of included participants was higher than 18 years [19, 21, 30, 33, 36, 38]. The results reported in this review, therefore, do not solely represent the pediatric cancer population, but also to some extent young adults with cancer. Even though this review has limitations, they have been counteracted by the search methods being carefully implemented by a research librarian and with the use of critical appraisal tools to assess the methodological quality of the articles. The methodological quality of the studies varied between medium and high. One study was classified as low quality and was excluded from further analysis.

### Implication for practice and further research

The review and meta-analysis indicate that CAM and more specifically acupuncture treatments have a positive effect in the treatment of vomiting and nausea associated with cancer treatment in children and young adults. Acupuncture is considered less invasive, and less liable to adverse effects [42].

### Implication for research

Even though the meta-analyses show a positive effect of acupuncture on chemotherapy-induced nausea and vomiting in children and young adults, it is important to conduct further research to establish if some forms of acupuncture (acupressure, needle acupuncture, or laser acupuncture) are more effective than others. While hypnosis did not show a significant effect in the meta-analysis, it is important to conduct more RCTs with large sample sizes to further determine the effect of hypnosis on CINV. It is also important to expand the research on different CAM modalities that are being used to treat cancer treatment-induced symptoms in children. Future research should focus on conducting RCTs with larger samples size to further establish the effect of (the) CAM therapies. Also, RCTs should more diligently report whether there were any adverse effects from the therapies studied. Although some studies in this review reported adverse effects, the majority did not. Adverse effects are underreported in CAM research, the majority of the studies in this review (*n* = 15, 71%) did not collect any safety data (see Table 1). The report of adverse effects is important to establish the safety of the CAM therapies especially related to interactions with conventional chemotherapy treatment. It is also important for the researchers to carefully design the studies to use standard measurements of the outcomes to enable comparison to other studies in the area.

## Conclusion

This systematic review and meta-analysis suggest a significant overall effect of CAM (including acupuncture and hypnosis only) on CINV among children and young adults compared to the control interventions. The use of acupuncture might be considered as a complementary measure to help children cope with nausea and vomiting. CAM modalities such as acupuncture or hypnosis can easily be implemented in healthcare settings, however more rigorous trials are needed, and long-term effects should be investigated before it is recommended for clinical practice. To further establish the safety of CAM modalities and the findings of this review, it is imperative to conduct more research on different CAM modalities.

## Supplementary Information

**Additional file 1.** Literature review search strategy.

**Additional file 2.** PRISMA 2020 checklist CAM children.

## Data Availability

All data generated or analyzed during this study are included in this published article [and its supplementary information files].

## Abbreviations

ALL: Acute lymphoblastic leukemia
AST: Aspartate amino transferase
ALT: Amino alanine transferase
HEC: Highly melogenic chemotherapy
CAM: Complementary and alternative medicine
CIV: Chemotherapy-induced vomiting
CIN: Chemotherapy-induced nausea
CINV: Chemotherapy-induced nausea-vomiting:
HICs: High-income countries
NR: Not reported in the publication
JBI: Joanna Briggs Institute
PRISMA: Preferred Reporting Items for Systematic review and Meta-Analyses
RCTs: Randomized controlled trials
SMD: Standardized mean difference
SUMARI: System for the Unified Management, Assessment, and Review of Information

## Footnotes

1 * = truncation—the search is done for the beginning of the word and all possible endings.

## References

[1] Cancer Registry of Norway. Cancer in Norway 2020. Cancer incidence, mortality, survival and prevalence in Norway. Oslo: Cancer Registry of Norway. https://healthtalkweb.s3.amazonaws.com/documents/Cancer_in_Norway_2020.pdf; 2020.

[2] Lam CG, Howard SC, Bouffet E, Pritchard-Jones K (2019). Science and health for all children with cancer. Science.

[3] Hedén L, Pöder U, von Essen L, Ljungman G. Parents’ Perceptions of Their Child’s Symptom Burden During and After Cancer Treatment. J Pain Symptom Manage. 2013;46(3):366–75. 10.1016/j.jpainsymman.2012.09.012 23498966

[4] Stub T, Kristoffersen AE, Overvåg G, Jong MC (2020). An integrative review on the information and communication needs of parents of children with cancer regarding the use of complementary and alternative medicine. BMC Complement Med Ther.

[5] Radossi AL, Taromina K, Marjerrison S, Diorio CJ, Similio R, Njuguna F (2018). A systematic review of integrative clinical trials for supportive care in pediatric oncology: a report from the International Society of Pediatric Oncology. T&CM collaborative Support Care Cancer.

[6] Complementary, Alternative, or Integrative Health: What’s In a Name? National Center for Complementary and Integrative Health2018 [updated 2018. Available from: https://www.nccih.nih.gov/health/complementary-alternative-or-integrative-health-whats-in-a-name.

[7] Hawkins J, Hires CY, Dunne EW, Keenan LA (2020). Aromatherapy reduces fatigue among women with hypothyroidism: A randomized placebo-controlled clinical trial. Journal of Complementary & Integrative Medicine.

[8] Gottschling S, Reindl TK, Meyer S, Berrang J, Henze G, Graeber S (2008). Acupuncture to alleviate chemotherapy-induced nausea and vomiting in pediatric oncology - A randomized multicenter crossover pilot trial. Klin Padiatr.

[9] Reindl TK, Geilen W, Hartmann R, Wiebelitz KR, Kan G, Wilhelm I (2006). Acupuncture against chemotherapy-induced nausea and vomiting in pediatric oncology. Interim results of a multicenter crossover study. Sup Care Cancer.

[10] Bishop FL, Prescott P, Chan YK, Saville J, von Elm E, Lewith GT (2010). Prevalence of complementary medicine use in pediatric cancer: a systematic review. Pediatrics.

[11] Krogstad T, Nguyen M, Widing E, Toverud EL (2007). Children with cancer and their use of natural products. Tidsskr Nor Laegeforen.

[12] Page MJ, McKenzie JE, Bossuyt PM, Boutron I, Hoffmann TC, Mulrow CD, et al. The PRISMA 2020 statement: an updated guideline for reporting systematic reviews. Bmj. 2021;372. 10.1136/bmj.n71 PMC8005924 33782057

[13] Ouzzani M, Hammady H, Fedorowicz Z, A E (2016). Rayyan—a web and mobile app for systematic reviews. Syst Rev.

[14] Higgins JPT, Green S (2008). Cochrane handbook for systematic reviews of interventions.

[15] Peters M, Godfrey C, McInerney P, Soares C, Khalil H, Parker D. The Joanna Briggs Institute reviewers' manual 2015: methodology for JBI scoping reviews. 2015.

[16] Stub T, Kristoffersen AE, Overvåg G, Jong MC, Musial F, Liu J (2020). Adverse effects in homeopathy.

[17] Review Manager (RevMan) [Computer program] Version 5.4. The Cochrane Collaboration, 2020 2020.

[18] El-Housseiny AA, Saleh SM, El-Masry AA, Allam AA. The effectiveness of vitamin “E” in the treatment of oral mucositis in children receiving chemotherapy. J Clin Pediatr Dent. 2007;31(3):167–70. 17550040

[19] Jones E, Isom S, Kemper KJ, McLean TW (2008). Acupressure for chemotherapy-associated nausea and vomiting in children. J Soc Integr Oncol.

[20] Khurana H, Pandey RK, Saksena AK, Kumar A (2013). An evaluation of Vitamin E and Pycnogenol in children suffering from oral mucositis during cancer chemotherapy. Oral Dis.

[21] Pillai AK, Sharma KK, Gupta YK, Bakhshi S (2011). Anti-emetic effect of ginger powder versus placebo as an add-on therapy in children and young adults receiving high emetogenic chemotherapy. Pediatr Blood Cancer.

[22] Wada M, Nagata S, Saito M, Shimizu T, Yamashiro Y, Matsuki T (2010). Effects of the enteral administration of Bifidobacterium breve on patients undergoing chemotherapy for pediatric malignancies. Support Care Cancer.

[23] Abdulah DM, Abdulla BMO (2018). Effectiveness of group art therapy on quality of life in paediatric patients with cancer: A randomized controlled trial. Complement Ther Med.

[24] Ghezelbash S, Khosravi M (2017). Acupressure for nausea-vomiting and fatigue management in acute lymphoblastic leukemia children. Journal of Nursing & Midwifery Sciences.

[25] Nguyen TN, Nilsson S, Hellstrom AL, Bengtson A (2010). Music therapy to reduce pain and anxiety in children with cancer undergoing lumbar puncture: a randomized clinical trial. J Pediatr Oncol Nurs.

[26] Consolo LZZ, Melnikov P, Consolo FZ, Nascimento VA, Pontes JCDV (2013). Zinc supplementation in children and adolescents with acute leukemia. Eur J Clin Nutr.

[27] Jacknow DS, Tschann JM, Link MP, Boyce WT (1994). Hypnosis in the prevention of chemotherapy-related nausea and vomiting in children: a prospective study. J Dev Behav Pediatr.

[28] Zeltzer LK, Dolgin MJ, LeBaron S, LeBaron C (1991). A randomized, controlled study of behavioral intervention for chemotherapy distress in children with cancer. Pediatrics.

[29] Rathe M, De Pietri S, Sangild PT, Husby S, Frandsen TL, Wehner SW (2018). The use of bovine colostrum against chemotherapy-induced gastrointestinal toxicity in children with acute lymphoblastic leukemia: A double-blind placebo-controlled randomized trial. Pediatr Blood Cancer.

[30] Tomazevic T, Jazbec J (2013). A double blind randomised placebo controlled study of propolis (bee glue) effectiveness in the treatment of severe oral mucositis in chemotherapy treated children. Complement Ther Med.

[31] Dupuis LL, Kelly KM, Krischer JP, Langevin AM, Tamura RN, Xu P (2018). Acupressure bands do not improve chemotherapy-induced nausea control in pediatric patients receiving highly emetogenic chemotherapy: A single-blinded, randomized controlled trial. Cancer.

[32] Evans A, Garretson C, Pedroja E, Malvar J, Margol A, Sposto R (2016). The use of aromatherapy to reduce chemotherapy-induced nausea in children with cancer; A randomized, double blind, placebo controlled trial. Neuro-Oncol.

[33] Ladas EJ, Kroll DJ, Oberlies NH, Cheng B, Ndao DH, Rheingold SR (2010). A randomized, controlled, double-blind, pilot study of milk thistle for the treatment of hepatotoxicity in childhood acute lymphoblastic leukemia (ALL). Cancer.

[34] Nguyen TN, Nilsson S, Hellström A, Bengtson A (2010). Music therapy to reduce pain and anxiety in children with cancer undergoing lumbar puncture: a randomized clinical trial. J Pediatr Oncol Nurs.

[35] Varejao CDS, Santo F (2019). Laser Acupuncture for Relieving Nausea and Vomiting in Pediatric Patients Undergoing Chemotherapy: A Single-Blind Randomized Clinical Trial. J Pediatr Oncol Nurs.

[36] Ward E, Smith M, Henderson M, Reid U, Lewis I, Kinsey S (2009). The effect of high-dose enteral glutamine on the incidence and severity of mucositis in paediatric oncology patients. Eur J Clin Nutr.

[37] Yeh CH, Chien LC, Chiang YC, Lin SW, Huang CK, Ren D (2012). Reduction in nausea and vomiting in children undergoing cancer chemotherapy by either appropriate or sham auricular acupuncture points with standard care. J Altern Complement Med.

[38] Evans A, Malvar J, Garretson C, Pedroja Kolovos E, Baron NM (2018). The Use of Aromatherapy to Reduce Chemotherapy-Induced Nausea in Children With Cancer: A Randomized, Double-Blind. Placebo-Controlled Trial J Pediatr Oncol Nurs.

[39] Tsukinoki R, Murakami Y (2013). Non-communicable disease epidemic: epidemiology in action (EuroEpi 2013 and NordicEpi 2013): Aarhus, Denmark from 11 August to 14 August 2013. Eur J Epidemiol.

[40] O’Regan D, Filshie J. Acupuncture and cancer. Auton Neurosci. 2010;157(1):96–100. 10.1016/j.autneu.2010.05.001 20605536

[41] Flank J, Robinson PD, Holdsworth M, Phillips R, Portwine C, Gibson P (2016). Guideline for the treatment of breakthrough and the prevention of refractory chemotherapy-induced nausea and vomiting in children with cancer. Pediatr Blood Cancer.

[42] Vincent C (2001). The safety of acupuncture. BMJ (Clinical research ed).

[43] Chokshi SK, Ladas EJ, Taromina K, McDaniel D, Rooney D, Jin Z, et al. Predictors of acupuncture use among children and adolescents with cancer. Pediatr Blood Cancer. 2017;64(7):e26424. 10.1002/pbc.26424 28176457

[44] Yeh CH, Zhao TY, Zhao MD, Wu Y, Guo YM, Pan ZY (2019). Comparison of effectiveness between warm acupuncture with local-distal points combination and local distribution points combination in breast cancer-related lymphedema patients: a study protocol for a multicenter, randomized, controlled clinical trial. Trials.

[45] Cohen J (1988). Statistical power analysis for the behavioral sciences.

[46] Qiu T, Men P, Xu X, Zhai S, Cui X. Antiemetic regimen with aprepitant in the prevention of chemotherapy-induced nausea and vomiting: An updated systematic review and meta-analysis. Medicine (Baltimore). 2020;99(33). 10.1097/MD.0000000000021559 PMC7437786 32872006

[47] Yuan D-M, Li Q, Zhang Q, Xiao X-W, Yao Y-W, Zhang Y (2016). Efficacy and safety of neurokinin-1 receptor antagonists for prevention of chemotherapy-induced nausea and vomiting: systematic review and meta-analysis of randomized controlled trials. Asian Pac J Cancer Prev.

[48] Fugetto F, Filice E, Biagi C, Pierantoni L, Gori D, Lanari M (2020). Single-dose of ondansetron for vomiting in children and adolescents with acute gastroenteritis—an updated systematic review and meta-analysis. Eur J Pediatr.

[49] Tricco AC, Blondal E, Veroniki AA, Soobiah C, Vafaei A, Ivory J (2016). Comparative safety and effectiveness of serotonin receptor antagonists in patients undergoing chemotherapy: a systematic review and network meta-analysis. BMC Med.

[50] Lau Moon Lin M, Robinson PD, Flank J, Sung L, Dupuis LL (2016). The Safety of Metoclopramide in Children: A Systematic Review and Meta-Analysis. Drug Safety.

[51] Bishop FL, Prescott P, Chan YK, Saville J, von Elm E, Lewith GT (2010). Prevalence of Complementary Medicine Use in Pediatric Cancer: A Systematic Review. Pediatrics.

[52] Diorio C, Kelly KM, Afungchwi GM, Ladas EJ, Marjerrison S. Nutritional traditional and complementary medicine strategies in pediatric cancer: A narrative review. Pediatr Blood Cancer. 2020;67. 10.1002/pbc.28324 32614139

[53] Kelly KM (2004). Complementary and alternative medical therapies for children with cancer. Eur J Cancer.

[54] Cheng C-W, Fan W, Ko S-G, Song L, Bian Z-X (2010). Evidence-Based Management of Herb-Drug Interaction in Cancer Chemotherapy. EXPLORE.

[55] Sagar SM (2009). Evidence-based clinical practice guidelines for integrative oncology: complementary therapies and botanicals (2009). J Soc Integr Oncol.

[56] Kanitz JL, Camus MEM, Seifert G (2013). Keeping the balance – an overview of mind–body therapies in pediatric oncology. Complement Ther Med.

[57] Jong MC, Boers I, van Wietmarschen H, Busch M, Naafs MC, Kaspers GJL (2020). Development of an evidence-based decision aid on complementary and alternative medicine (CAM) and pain for parents of children with cancer. Support Care Cancer.

[58] Facchini M, Ruini C. The role of music therapy in the treatment of children with cancer: A systematic review of literature. Complement Ther Clin Pract. 2021;42. 10.1016/j.ctcp.2020.101289 33316592

[59] Joseph PD, Craig JC, Caldwell PHY (2015). Clinical trials in children. Br J Clin Pharmacol.

[60] Bond MC, Pritchard S (2006). Understanding clinical trials in childhood cancer. Paediatr Child Health.

[61] Pirotta M (2007). Towards the application of RCTs for CAM.

