# Supplementary material for: Complementary and alternative medicine modalities used to treat adverse effects of anti-cancer treatment among children and young adults: a systematic review and meta-analysis of randomized controlled trials
Source: BMC Complement Med Ther. 2022 Apr 2;22:97. doi: 10.1186/s12906-022-03537-w (PMC8976304; doi:10.1186/s12906-022-03537-w)
Supplement: Supplementary file 1 — Additional file 1. Literature review search strategy. [file 12906_2022_3537_MOESM1_ESM.docx]

**Literature Review**

**AIM:** CAM therapies/modalities used to treat adverse effects/side effects of conventional cancer treatment among children with cancer

**Population** – Children the ever had cancer between the ages of 0-18 years of age

**Intervention –** CAM therapies/modalities

**Comparison –** Conventional medicine, no treatment, other CAM treatments, placebo

**Outcome –** Reduction/improvement of side-effects/adverse effects

**Studies –**randomized control trials

**Filter: t**imeframe from 1990’s to present, humans

**Languages:** Scandinavian languages, German, Dutch, English, Spanish

**Databases**: AMED, CINAHL, EMBASE, PubMed, PsycINFO, the Cochrane Library.

| Conventional Cancer Treatment | CAM treatment/ modalities | Patients | Adverse Effects |
| --- | --- | --- | --- |
| - Neoplasm - Leukemia - Lymphoma/soft tissue sarcoma - Pediatric cancer - Pediatric oncology - Integrative oncology - Cancer treatment - Childhood cancer - Pediatric palliative care | - CAM modalities - CAM treatment - CAM - Integrative medicine - Complementary medicine - Alternative medicine - Unconventional medicine - Spiritual healing/faith healing | - Children - Child* - Infant - Adolescent - Juvenile - Pediatric - Puberty - Young adults - Young person - Teen* - Childhood - Toddler | - Side effects - Adverse effects - Safety - Risk factors - Harm - Adverse reactions - Indirect/direct risks - Adverse drug reaction - Symptom management - Hopelessness/suffering |

**MESH/Medline (Pubmed) og AMED**

| Exp Neoplasms | Exp Complementary Therapies  Exp Integrative medicine | Exp Child  Exp Adolescent exp Young adult exp Infant | Adverse effects.fs (subheading, fs)  Drug-Related Side Effects and Adverse Reactions  exp Adverse Drug Reaction Reporting Systems | exp randomized controlled trial  Cross-Sectional Studies  Cohort Studies  qualitative research  exp Interview  exp Observational Study |
| --- | --- | --- | --- | --- |

Emtree headings (Embase)

| Exp Neoplasms | exp Complementary Therapies Exp Integrative medicine | Exp Child Exp Adolescent exp Young Adult/ exp Infant/ | Exp Adverse event side effect.fs  side effect.fs.  exp "Drug-Related Side Effects and Adverse Reactions"/  exp Adverse Drug Reaction Reporting Systems/ | exp randomized controlled trial  Cross-Sectional Studies  Cohort Studies  qualitative research  exp Interview  exp Observational Study |
| --- | --- | --- | --- | --- |

Cinahl headings

| Exp Neoplasms | Exp Alternative therapies | Exp Child  Exp Adolescence | Exp Adverse drug event | Exp Randomized controlled trials  Exp Nonexperimental studies  Exp Qualitative studies |
| --- | --- | --- | --- | --- |

PsycInfo Thesaurus

| Exp Neoplasms |  |  | exp "Side Effects (Drug)" | exp randomized controlled trial  Cohort Studies  qualitative research |
| --- | --- | --- | --- | --- |
